# Abstract of the Proceedings of the Buffalo Medical Association

**Published:** 1864-10

**Authors:** Joseph A. Peters

**Affiliations:** Secretary


ART. II.—*Abstract of the Proceedings of the Buffalo Medical Association.*

TUESDAY EVENING, September 5, 1864.

Society met pursuant to adjournment, the President, Dr. Samo, in the chair. Present—Drs. White, Rochester, Lockwood, Strong, Miner, Wyckoff, Gay, Shaw, Ring, Cronyn, Dayton, Congar, Johnson and Peters.

*Dr. Gay* proposed for membership the name of Dr. Hauenstein. Laid over under the rules.

*Dr. Gay* presented the following paper on Erysipelas:

The unusual prevalence of abscess, the tendency to the formation of pus depots, and the large number of cases of cutaneous and cellulo-cutaneous forms of erysipelas must have been observed and attracted the attention of all during the past season, especially during the early spring. Indeed so great was the tendency to erysipelas and pyæma after surgical operations that, in one of our hospitals at least, operations were for a time interdicted, death after operations seeming to be the rule, and recovery the exception, despite the utmost precaution and skill on the part of the surgeon.

I have witnessed in three instances a considerable abscess result from puncture made with the hypodermic syringe, but attributed at the time, the formation of the abscess, more to the preparation of morphia used than to the puncture made with the syringe. It is well understood that the acetate, instead of the sulphate of morphia, should be used in hypodermic medication, the sulphuric acid of the sulphate being capable of exciting inflammation when injected into the areolar tissue. Yet I am in doubt still, whether the abscesses in question were, or were not, caused by the preparation of morphia used, or whether the puncture itself was not sufficient to have caused them.

I have a case in point which, so far as it goes, impairs the hypothesis just alluded to. In order to determine the nature of a swelling a very small exploring trocar was introduced which in two or three days was followed by suppuration, the pus being independent of and superficial to the tumor.

In view of these facts, I have thought it would not be wholly without interest to bring the subject of abscess before the Society at this time, especially that form of abscess so commonly met with, and which has been apparently modified by an erysipelatous dyscrasia, detailing a few representative or typical cases with the ultimate object to indicate the best mode of treatment to be adopted.

Independent of the intention just mentioned, it is believed that the cases which will be brought forward possess some intrinsic interest *per se*. But before I c me to the cases, I will premise, by calling attention to the fact that little has been written or published, either in our current medical literature or standard works upon the topic under consideration, perhaps the subject demands no more notice. Miller's Surgery contains four pages upon the subject of Phlegmonous Erysipelas and its treatment. Gross and Druitt have each but a single paragraph. It would appear, therefore, that a surgical hiatus exists, to fill up which, some writer might profitably occupy his time.

*Case 1st.*—Mrs. H., German, aged 30 years, previous health good, was attacked with rigors, followed with pain at a point near and posterior to the right breast, after the lapse of two weeks, although the pain had been continuous, there was no perceptible swelling or fluctuation, but the whole side was somewhat fuller than its opposite.

For local treatment, poultices and blisters had been used, and the patient sustained by good nourishment and tonics. The general symptoms, to wit, extreme prostration, frequent pulse, and profuse perspiration, conjoined to the local symptoms afforded sufficient evidence of the existence of pus, and the propriety of giving exit to it; consequently on February 13th, two weeks after the attack, a deep incision was made directly over the seat of pain, and the grooved director pushed down through, in an oblique direction, nearly its entire length, before the walls of the abscess were penetrated; the opening thus made was dilated, and one pint or more of pus obtained. A tent was introduced to the bottom of the wound, and renewed every day for a week, when the discharge ceased, and the patient pronounced convalescent in four weeks from the attack.

*Case 2d.*—Mr. H., aged 33 years, husband of the former, was taken sick about one week before the convalescence of the wife, with pain in nearly the same locality, upon the right chest. The general and local symptoms not much unlike those of the other case. The opening of the abscess was not made so early, the prostration was greater, and symptoms more imminent.

March 19th, three weeks after the attack, a deep opening was made down through the entire thoracic muscles, and one pint of pus discharged; kept the wound open with the tent.

April 10th, not mnch improvement. Made another incision and obtained about one-half pint of very offensive pus.

15th, decidedly improved, and in a few days was convalescent. Duration of disease seven weeks. These two patients were visited by myself, in consultation with Dr. Hauenstein, who has kindly permitted me to report them.

*Case 3d.*—Mr. P., American, aged 38 years, was called February 21st. Patient complained of sore throat. On examination, found the pharynx inflamed. Made application of sol. argt. nitras. After each application the inflammation seemed to be aggravated. I consequently desisted from any further application, except a solution of morphia.

The case gradually grew worse, until deglutition was extremely painful. The inflammation assumed a migratory character, beginning with the pharynx, which soon presented a healthful appearance, traveling downwards to the larynx, remaining for a time, leaving it, in its turn, with a healthy aspect, so far as I was able to ascertain.

Yet, with the improvement of the mucus membrane, deglutition was becoming more, and still more, painful. I was therefore led to attribute the pain to the action of the muscles of deglutition. Tenderness, upon pressure, was now apparent at the left of larynx; considerable fullness over the entire left side of the neck. Poultices and blisters were used, but nothing gave relief but full doses of morphia.

March 7th, made an incision at the left and by the side of the larynx as deep as I deemed it safe to go, then with the grooved director penetrated the walls of the abscess, dilated the opening and obtained a large quantity of pus.

March 11th. Discharge ceased. Made another opening, discharged freely; used the tent.

March 14th. Discharge entirely ceased, and in a few days the patient was convalescent. Duration of sickness three weeks.

It was my fortune, during the past season, to see a half-dozen other cases of the same character precisely, two of which occurred in the persons of husband and wife, the abscesses locating respectively beneath the right pectoral muscles and the deep fascia of the thigh, but as they can contribute nothing toward furthering the object of this paper, they are omitted.

The assault, however, of this disease upon two members of the same family, consecutively suggests the question of contagion. I cannot, however, in any sense, regard the disease as contagious, unless there be real contact of pus with an abraded surface. I must rest satisfied, therefore, in the belief that the same sanitary and hygienic conditions, as bad air, blood, etc,, which incited to the disease in the one case, would, when superadded to excessive anxiety and interrupted sleep, predispose to and develop in him, who continually watches by the bed-side of the sick, the same form of disease.

I have taken the liberty of calling these reported cases of disease, Phlegmonoid Erysipelas. They might be thought to be nothing more than common abscesses, but they differ materially from simple and pure abscess. All the conditions of simple abscess were absent, except one, viz.: the formation of pus. Added to the negative evidence of the erysipelatous character of these abscesses, there is the positive evidence existing in the fact of the prevalence of erysipelas, that during the progress of Case No. 3, there was a case of cutaneous erysipelas in the same family. The sudden onset of pain, the chills and active febrile movement, the deep-seated inflammation, its duration and the unmistakable tendency to blood poison—all contribute to render the differential diagnosis easy.

Phlegmonous differs from phlegmonoid erysipelas in this, that in the former the inflammation attacks the subcutaneous cellular tissue, while in the latter the inflammation is more profound.

As an example of the former, a case may be cited: A person is attacked with inflammation of the finger or hand, which, traveling upwards, perhaps reaches the shoulder or it may be confined to the hand or fore-arm, the pus quickly pointing to the surface and suppurating, but sometimes requiring incisions to hasten the suppurative process or even to give exit to the pus. The two forms of abscess differ materially.

The indications of treatment are plain, to wit: relief of pain and the exit of pus. To this end anodynes, poultices, blisters, wet and dry cupping and the knife are required. It is somewhat questionable whether anything can be of much service but the knife.

I desire to point out what I conceive to be a therapeutical fallacy in the treatment of this affection. It is in the use of discutients and sorbefacients, with the hope of repelling the inflammation or promoting resolution or absorption. These desirable results cannot be accomplished. Much time may be needlessly wasted in attempts to this end, and the life of the patient imperiled. I cannot, in too strong language, denounce the use of iodine, yet how naturally it suggests itself, and how eagerly is it seized upon as the infallible agent. I believe that theory, practice and common sense, all combine to condemn it. An effort should unquestionably be made to invite pus to the surface, and the employment of suitable constitutional remedies should be had recourse to, to keep the pus out of the blood. But the knife should be chiefly relied upon, and used as early as possible, never waiting to detect fluctuation, for by too much delay, one deep abscess may degenerate into many fresh ones, occupying parts remote from the original or primary abscess, or pus might be absorbed into the blood, ending the case in pyaemia and death. If pus can be obtained early and abundantly, it becomes impossible for the patient to die of this disease if he be properly sustained.

*Dr. White* would like to call up a subject which had possessed great interest for himself, and he thought could not fail to interest others. He referred to the growth and involution of the uterus incident to pregnancy and parturition, that wonderful process by which an organ weighing not more than ten or twelve drachms, and of a capacity of less than one cubic inch, in the virigin increased to a weight of twenty or thirty ounces, and a capacity of 450 cubic inches in the pregnant woman, and again decreased to nearly a weight of two and a half ounces, and correspondingly small measurements. Without going into any discussion on the subject he would give it as his belief that the uterus consisted in its normal state of nascent muscle, which becomes developed into striated muscular structure, and is again reduced in size by a process of fatty degeneration and absorption. Interesting as this process was, however, he wished to speak more particularly of the process of degeneration, or involution, as it is called, and of its variations. It presented two variations from the normal standard which constituted diseases, viz: sub-involution, when the process was arrested before the normal standard was reached, and super-involution, when the process had gone on to too great an extent, and the uterus was reduced below its normal size. The former was much the more frequent, and is often met with after abortions and after frequent pregnancies. The second variation, super-involution, or atrophy, was much more rare, and so far as he knew was not recognized or treated of by authors of systematic treatises on obstetrics. Had notes of three cases in the last two or three years which he would lay briefly before the Association, for the purpose of calling attention to this condition.

*Case* 1*st.*—A lady, aged 18, was sent to him by Dr. Potter, from Genesee county. The patient had had a severe labor, and was delivered after protracted efforts with the instruments of a dead child. No laceration followed; she was afflicted with severe inflammation within the pelvis, and convalesced very slowly. Saw her about eight or nine months after delivery; the menstrual molimen was present, but there was no flow; she suffered some pain at each menstrual epoch, but her general health seemed good. On examination he found the vagina nearly closed with adhesions; divided them, dilated the vagina, and on reaching the uterus found only vestiges of the os which was closed, the organ being as small as in a girl of twelve years, as felt through the walls of the rectum. In December, 1862, in presence of Drs. Moore, Eastman and others, he divided the tissues for one and one-half inches in the line of the cavity of the neck and body, and inserted a sponge tent. Had inserted sponge tents from time to time since. The uterus is still small, the molimen is very marked, great pain being suffered, but no flow making its appearance. Her general health is good, except at the menstrual period. Did not yet despair of eventually restoring the functions of the organ, and proposed inserting a galvanic pressary.

*Case 2d.*—A lady from Michigan, aged 26. The previous history was incomplete; the patient had not menstruated for four years, but whether she was regular before that, or not, could not be ascertained. Her general health had suffered much, and she complained of great pain at the appearance of the menstrual molimen. On examination, in November, 1863, he found he could not pass the smallest probe through the neck of the uterus. Guided by a probe and the direction of the cavity of the neck, he made an opening of an inch and a half or more, dilated it with tents, and then introduced a short galvanic pessary. She returned in three months improved in health, and had had slight sanguinolent discharges at each monthly period. In August she had so far improved as to have the menses pretty well, and the uterus has perceptibly increased in size. Continued the use of the pessary, with air, tonics, iron, etc.

*Case 3d.*—Aged 34, from Pennsylvania. Had a severe labor in April last, since which time she has not menstruated. Found the vagina, on examination in July, nearly closed. Together with Dr. Congar, he dilated the vagina fully with sponge tents, but so little time had elapsed since labor, that he had concluded to await natural efforts for a few months.

In conclusion, he would say that the appropriate treatment, as it seemed to him, was that which he had indicated, viz.: the breaking up of all adhesions by the use of sponge tents, the giving of tonics, iron, etc., fresh air, nutritious diet, and the stimulating the uterus to act by the use of the galvanic pessary.

*Dr. Gay* called attention to the plant presented by Dr, Lockwood at the last meeting. Had examined it, and found it to be not Apocynum, but Asclepias. Exhibited specimens of both plants.

*Dr. Lockwood* did not claim to be a systematic botanist, but had gathered and used the root of the plant he presented for the past thirty years, and had always considered it Apocynum Cannabiuum. Moved that a committee, to consist of Drs. Gay, Strong and Peters, be appointed, to report on the subject at the next meeting. Carried.

Reports on prevailing diseases being called for, Dr. Wyckoff mentioned having seen a good deal of typhoid fever and dysentery.

*Dr. Dayton* had seen considerable intermittent.

*Dr. Wyckoff* had seen several cases of typhoid fever. In one family, where he attended, had seen seven cases, six being down at one time. The youngest children had diarrhœa and dysentery, with discharges of bloody follicles, febrile re-action, etc. He considered the same influence had been at work on the whole family. Could find no local cause for it.

Society adjourned.

JOSEPH A. PETERS, Secretary.

